# Targeting the Stress System During Gestation: Is Early Handling a Protective Strategy for the Offspring?

**DOI:** 10.3389/fnbeh.2020.00009

**Published:** 2020-01-31

**Authors:** Valentina Castelli, Gianluca Lavanco, Anna Brancato, Fulvio Plescia

**Affiliations:** ^1^Department of Biomedicine, Neuroscience and Advanced Diagnostics, University of Palermo, Palermo, Italy; ^2^INSERM U1215, Neuro Centre Magendie, Bordeaux, France; ^3^University of Bordeaux, Bordeaux, France; ^4^Department of Biomedical and Biotechnological Sciences, Section of Pharmacology, University of Catania, Catania, Italy; ^5^Department of Health Promotion, Mother and Child Care, Internal Medicine and Medical Specialties “Giuseppe D’Alessandro”, University of Palermo, Palermo, Italy

**Keywords:** prenatal exposure, glucocorticoid, early handling, stress reactivity, depressive-like behavior, emotionality

## Abstract

The perinatal window is a critical developmental time when abnormal gestational stimuli may alter the development of the stress system that, in turn, influences behavioral and physiological responses in the newborns. Individual differences in stress reactivity are also determined by variations in maternal care, resulting from environmental manipulations. Despite glucocorticoids are the primary programming factor for the offspring’s stress response, therapeutic corticosteroids are commonly used during late gestation to prevent preterm negative outcomes, exposing the offspring to potentially aberrant stress reactivity later in life. Thus, in this study, we investigated the consequences of one daily s.c. injection of corticosterone (25 mg/kg), from gestational day (GD) 14–16, and its interaction with offspring early handling, consisting in a brief 15-min maternal separation until weaning, on: (i) maternal behavior; and (ii) behavioral reactivity, emotional state and depressive-like behavior in the adolescent offspring. Corticosterone plasma levels, under non-shock- and shock-induced conditions, were also assessed. Our results show that gestational exposure to corticosterone was associated with diminished maternal care, impaired behavioral reactivity, increased emotional state and depressive-like behavior in the offspring, associated with an aberrant corticosterone response. The early handling procedure, which resulted in increased maternal care, was able to counteract the detrimental effects induced by gestational corticosterone exposure both in the behavioral- and neurochemical parameters examined. These findings highlight the potentially detrimental consequences of targeting the stress system during pregnancy as a vulnerability factor for the occurrence of emotional and affective distress in the adolescent offspring. Maternal extra-care proves to be a protective strategy that confers resiliency and restores homeostasis.

## Introduction

Numerous studies across a wide range of species have shown that prenatal exposure to different conditions such as infections, nutritional deficiencies, teratogenic substances, and emotional distress, predisposes the newborns to a spectrum of different disorders characterized by deficits in cognitive functioning, motor, and visuospatial abilities and to the genesis of chronic systemic diseases (Cannizzaro et al., 2002, 2005, 2006b, 2007, 2008; Hellemans et al., 2010; Leggio et al., 2014; Sarro et al., 2014; Martines et al., 2016; Moukarzel et al., 2018). Notably, maternal stress during pregnancy could dispose of the offspring toward vulnerability to neurobehavioral disorders. As mediators of the stress response, glucocorticoids are among the main primary programming factors conveying maternal stress to the fetus *via* the placenta (Zarrow et al., 1970; Schmidt et al., 2019), through the activation of the glucocorticoid receptors (GR), whose ontogenetic pattern has been detected in human from the early prenatal life stages (Kitraki et al., 1997; Diaz et al., 1998; Kemp et al., 2016). Thus, glucocorticoids, by a receptor-mediated regulatory role during ontogenic development, could affect normal brain neurogenesis (Cintra et al., 1993). In this regard, prospective animal- and retrospective human studies have revealed that antenatal glucocorticoid administration in late gestation can lead to lifelong alterations on brain structures and functionality and may produce long-lasting modifications in the maturation of the hypothalamic-pituitary-adrenal (HPA) axis (Heim et al., 1997; French et al., 1999, 2004; Sloboda et al., 2005; de Vries et al., 2007; Charil et al., 2010; Fowden and Forhead, 2015). Indeed, exposure to glucocorticoids during pregnancy, reducing negative-feedback on HPA axis, increases cortisol release in the progeny (Alexander et al., 2012): this leads to a slower recovery from stressors, reducing coping strategy in aversive situations (Welberg et al., 2001; Plescia et al., 2013). This evidence represents a key issue in the therapeutic administration of antenatal corticosteroids, which are commonly used when at risk of preterm delivery to ensure the survival of the preterm infant (Singh et al., 2012). Indeed, last-trimester administration of synthetic glucocorticoids also “programs” outcomes comparable to those elicited by prenatal stress in humans (Seckl et al., 2000). Accordingly, treating pregnant rodents with synthetic glucocorticoids leads to offspring with similar HPA axis- and behavioral changes as prenatally stressed offspring (Schmidt et al., 2019).

The gestational experiences may also affect the maternal-infant dyad (Tarullo et al., 2017; Reck et al., 2018). Indeed, pregnant women who experience social and emotional stress may divest themselves of maternal bonding (Baker et al., 2008; Azhari et al., 2019). Importantly, these conditions appear to have a major impact on child cognitive, emotional and physical development (Cogill et al., 1986; Bhagwanani et al., 1997; Smith et al., 2004). Alterations in maternal caregiving behavior after maternal stress, or exogenous administration of glucocorticoids, occur also in rodent models (Darnaudéry et al., 2004; Koehl et al., 2012; Jafari et al., 2017; Gemmel et al., 2018). For instance, acutely and repeatedly stressed dams spend less time in activities directed towards the pups rather than in self-oriented behaviors (Patin et al., 2002; Smith et al., 2004; Boero et al., 2018). After birth, the infant is dependent on the primary caregiver, not only for nursing and protection but also for the normal development of emotional behavior (Bella et al., 2018). Indeed, deficiency of motherly care during infancy affects the development of stress reactivity, contributing to the raising of the individual distinctness in emotional responses (Cannizzaro et al., 2005, 2006b). On the other hand, early handling procedures are able to significantly affect the development of the offspring’s emotional behavior and HPA axis physiology. In particular, extensive research has shown that brief periods of maternal separation of the pups during the nursing stage result in offspring decreased adrenal reactivity in response to stressors (Liu et al., 1997; Cannizzaro et al., 2005, 2006b, 2007; Plescia et al., 2014b), as well as fear-oriented behavior and emotionality (Cannizzaro et al., 2005, 2006b). The majority of these behavioral and neuroendocrine studies have been carried out on the adult progeny exposed to repeated prenatal stress. However, none of them has yet investigated the influence of the gestational exposure to corticosterone on emotional behaviors in adolescence, which emerges as a “critical” phase in the development of stress responsiveness (Cannizzaro et al., 2006b).

Thus, given these premises, the aim of the current study was to investigate the consequences of prenatal corticosterone exposure on maternal and offspring outcomes, during a timeframe when a relatively high expression of GR in multiple brain areas of the pups occurs (Cintra et al., 1993). In particular, we assessed maternal behavior, behavioral reactivity, emotionality and depressive-like behavior in the adolescent male offspring employing, respectively, the open field test (OFT), the acoustic startle reflex (ASR) and the forced swim test (FST). Offspring corticosterone plasma levels, under non-shock- and shock-induced conditions were evaluated as a measure of HPA axis activity. Early handling procedure, as a brief maternal separation, was also carried out as a putative protective strategy able to restore homeostasis.

## Materials and Methods

### Animals and Pharmacological Treatment

Wistar rats (Harlan, Udine, Italy) housed with free access to food and water were maintained on a 12 h on/off cycle (8:00–20:00 h) at a constant temperature (22 ± 2°C) and humidity (55 ± 10%). Pairs of primiparous females of 120 days of age were mated with one male of 150 days of age. The day on which sperm was detected in the vaginal smear was designed as gestational day (GD) 1. Pregnancy was determined by weighing and palpation. The pregnant dams’ weight on GD 14 was approximately 300 g. From GD 14 through GD 16, a period of time during which corticosterone can interact with GR expressed in the last week of gestation, the dams received a single daily subcutaneous injection of corticosterone (Ct; Sigma–Aldrich, Italy; 25 mg/kg) or vehicle (Vh; 100 mM DMSO in 0.9% saline solution) in a volume of 1 ml/kg. The pregnant dams were individually housed in standard rat cages (40 cm × 60 cm, 20 cm in height) for at least 7 days before delivery. All litters born within a 2-day period were reduced to ten pups (five males and five females) Forty male pups in total were used in our investigations; they were divided into the following experimental 10-rat (five rats per litter) groups: vehicle-non-handled (Vh); corticosterone-non-handled (Ct); vehicle-handled (Vh-H); corticosterone-handled (Ct-H). At weaning time, postnatal day (PND) 22, rats were randomly assigned two per cage accordingly to each experimental condition. The experiments were performed on adolescent rats—from PND 32 to 43. On the test day, each group of rats was brought into the laboratory and allowed to acclimate for at least 60 min prior to the experimental session. The experiments were performed in a sound isolated room between 9:00 and 14:00 and the animals were tested randomly, regardless of the group they belonged to. Animal performance during the different experimental sessions was recorded on the computer and then analyzed by an experimenter unaware of the different treatments. All the experiments were conducted in accordance with the regulations of the Committee for the Protection and Use of Animals of the University of Palermo, Italy, in accordance with current Italian legislation on animal experimentation (D.L. 26/2014) and the European Directive (2010/63/EU) on the care and use of laboratory animals. All efforts were made to minimize the number of animals used and possible distress.

### Early Handling and Pups Body Weight

Half of Ct- and Vh-treated litters remained undisturbed during the post-weaning period (i.e., non-handled, Ct and Vh groups), and half of prenatally Ct- and Vh-treated litters underwent early handling procedure (Ct-H and Vh-H groups), from PND 2 until PND 21. Early handling procedure consisted of removing the dam from the nest for 15 min during which she was temporarily placed in a separate cage. Simultaneously, pups were moved into a different room and individually placed into sawdust-containing small plastic cups for 15 min. In the end, mothers and pups were brought together in their home cages. Early handling procedure was performed in the same room, at the same time (10:00 h) and by the same experimenter. From PND 2 to PND 21 pups’ body weight was also evaluated.

### Maternal Behavior Assessment

Dam’s behavior in the presence of the offspring was assessed by direct periodic observations under undisturbed conditions in their home cages (Capone et al., 2005), from PND 2 to PND 21. Each animal was subjected to four assessments a day, during the diurnal time (9:00 am, 11:30 am, 01:30 pm, and 03:00 pm) when animals behave more maternally (Ader and Grota, 1970); instantaneous 20-s sampling was repeated three times at each time, for a total of 12 instantaneous observations per animal per day (3 observations × 4 times per day × 20 days = 240 observations per dam). The 20-s time of observation allows for an exact identification of the on-going behavioral patterns: retrieval, nursing (arched-back, blanket, passive), pup care (licking, anogenital licking), dam self-care (self-grooming, eating, drinking), and others (rearing, moving, resting, standing out of nest). Original data were recorded using dichotomous scores (0/1): score 0 was assigned when the behavior was not shown in the interval of observation; score 1 was assigned when the behavior was performed. Thus, a daily score ranged between 0 and 12. In order to gain a comprehensive framework of the behavioral measurements, a daily index of overall maternal behavior (MB-I) was calculated as follows: (maternal score) − (non-maternal score)/(maternal score) + (non-maternal score). The index ranges from −1 (totally non-maternal behaviors) to +1 (totally maternal behaviors; Brancato et al., 2016).

### Open Field Test

Locomotor activity and explorative behavior were assessed in the open-field arena with a contrast-sensitive, video tracking system, ANY MAZE (Ugo Basile, Gemonio, Italy), in a mean light intensity (100 lx) illuminated room (Brancato et al., 2014). The apparatus consisted in a square box (44 × 44 × 20 cm) and produced a quality-quantitative mapping of the ambulatory patterns, measuring simultaneously: total distance traveled (TDT) in centimeters, number of transition from peripheral to central squares of the arena (NCT) and amount of time spent on the central areas (ATC) in second. The 5-min recording and measurement of each experimental session started after 1-min habituation in the arena, to allow the rats to acclimatize, and was displayed on a personal computer (Cacace et al., 2011). The test was performed at PND 32.

### Acoustic Startle Reflex Test

The ASR provides a useful readout of the neural processing that might underpin an organism’s response to an emotional context or stressor (Hoffman, 2016; Hantsoo et al., 2018). The ASR response was measured using a Responder-X apparatus (Columbus Instruments, USA) at PND 34. The peak amplitude of the responses was recorded and displayed on a personal computer. A 10-min test session started by placing the rat in a 28 cm long, 16 cm wide, 15 cm high device with a stainless-steel grid floor, into a ventilated, sound-attenuated and darkroom, in which the animal was left undisturbed for the first 5 min period and was subsequently subjected to the startle stimulus for 5 min. The startle stimulus consisted of a 110 dB, 8 kHz tone superimposed on a continuous 50 dB white noise background; the stimulus duration was 200 ms, with a fixed 10-s interval. Sound levels in the test room were measured with a Bruel and Kjaer 2209 sound level meter. The maximum force exerted by the rat on-grid floor during the 200 ms period was designated as peak amplitude. The amplitude of ASR was measured in units, over the range of 60–550 g (1 unit = 2.1 g of force); maximum output was 255 units. The experimental session consisted of 10 trials.

### Forced Swim Test

We employed the FST as described by Porsolt et al. (1977) with some modifications, in order to test depressive-like behavior at PND 38. The test was composed of a pre-test stage (15 min) and, 24 h later, of a test stage (5 min), for both pre-test and test sessions, conducted under low illumination (12 lx), the animals were placed inside a transparent Plexiglas cylinder (50 cm high, 20 cm inside diameter) filled with tap clean water at 23 ± 1°C, adjusting the water depth according to the rat’s size, so that it cannot touch the bottom of the container with its hind legs (Yankelevitch-Yahav et al., 2015). A video camera was placed above the tank and connected to a video recorder to register each stage for subsequent scoring. An experimenter, unaware of the different treatments, scored the specific behavioral parameters from the videotape. Behavioral categories considered were as follows: immobility time, defined as floating in the water, making only the movement necessary to keep the head above water; swimming time, defined as making swimming motions and moving around the cylinder. Following either pre-test stage or test stage, the rats were dried with a towel and kept warm on a heating pad for 30 min in their home cages.

### Stress Procedure

At PND 43, rats from each experimental group were individually placed in a cage with an electrified grid floor through which shock could be delivered. The session started immediately after placing the rat into the shock-delivering apparatus. Rats (five per group) received an inescapable mild footshock (0.6 mA for 3 s) every 20 s, along 1 min. Control animals (non-stressed, five per group) were placed into the apparatus for the same time but were not shocked (Cannizzaro et al., 2006b).

### Plasma Corticosterone Assay

Rats were killed by decapitation 30 min after being placed into the shock-delivering apparatus. Trunk blood was collected into heparinized tubes. After centrifugation at 3,000 rpm at 4°C for 5 min, plasma samples were separated and stored at −80°C prior to assay. Plasma corticosterone concentration was assayed in duplicate using the RIA kit for rats (IDS Limited, Boldon, UK). The inter-and intra-assay coefficient of variation was 8% and 3% respectively, with a detection limit of 0.5 ng/mL. All measures were in the linear range of the standard curve (0.5–62.5 ng/mL).

### Statistical Analysis

Statistical data from bodyweight were carried out by a three-way ANOVA followed by Tukey’s test post-test (*α* = 0.05).

Statistical analysis of the data from the OFT, ASR, FST, maternal behavior scores and from non-shock- and shock-induced corticosterone plasma levels were carried out using a two-way ANOVA for unpaired measures. When necessary, *post hoc* comparisons were calculated with Tukey’s multiple comparison post-test (*α* = 0.05). Data are reported as mean ± SD. Statistical significance was set at *p* < 0.05.

## Results

### Pups Body Weight

**Figure 1:**
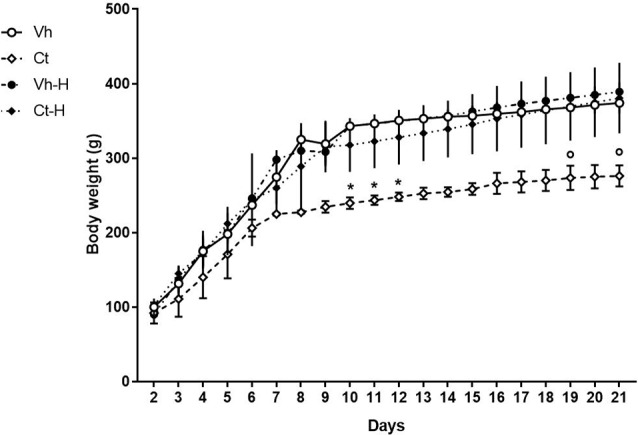
Graph showing the effect of prenatal corticosterone on body weight from postnatal day 2 until 21. Each value represents the mean ± SD of 10 rats. *p < 0.05 vs. Vh, °*p* < 0.05 vs. Vh-H.

Rats’ body weight was recorded from PND 2 to PND 21 in order to obtain data related to the influence of a single daily corticosterone administration and early handling procedure on weight gain during the pre-weaning period. No significant differences in number, weight, morbidity or mortality were observed among the different experimental groups. The results of a three-way ANOVA performed on body weight as a dependent variable, and days, prenatal corticosterone exposure and early handling as independent variables are shown in Table 1. The table indicates that: the factors days, prenatal corticosterone treatment, and early handling were significant. Moreover, the interaction between days- and prenatal treatment- with early handling was significant. The results of Tukey’s multiple comparisons test performed on each single day showed a reduction in body weight in Ct treated rats on days 10, 11 and 12 (*q* = 6.29, *p* = 0.04340; *q* = 6.25; *p* = 0.0463; *q* = 6.219, *p* = 0.0495), with respect to Vh groups; and a decrease in body weight on days 19 and 21 (*q* = 5.825, *p* = 0.0270; *q* = 6.341; *p* = 0.0380) in Ct with respect to Ct-H groups (Figure 1). No statistical difference was observed when Ct-H was compared to Vh and Vh-H groups.

**Table 1:** Pups body weight: results of three-way ANOVA performed on body weight as dependent variable and days (1), prenatal treatment with corticosterone (2), and early handling (3) as independent variables.

Source of Variation	DF	SS	MS	F	*P*-level
1-days	19	922,580	48,557	89.39	<0.001
2-prenatal treatment	1	54,686	54,686	100.7.83	<0.001
3-early handling	1	77,176	77,176	142.1.33	<0.001
1:2	19	9,384	492	0.9057	=0.5775
1:3	19	17,917	943	1.736.79.1	=0.0468
2:3	1	42,968	42,968	0.6584	<0.001
1:2:3	19	6,795	357.7		=0.8477
Residuals	80	43,457	543.2		

*Pups’ body weight (g) was expressed as the weight for the entire litter*.

### Dams Spontaneous Maternal Behavior

**Figure 2:**
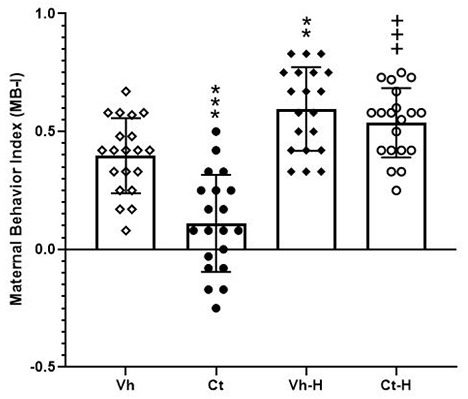
Maternal Behavior Index (MB-I). Influence of a daily 15-min early handling procedure on dams spontaneous behavior (retrieval, nursing, pup care, dam self-care). Each value represents the mean of ± SD of 20 measures. ***p < 0.001, **p < 0.01, vs. Vh, ^+++^*p* < 0.001 vs. Ct.

In order to evaluate the impact of gestational manipulation by corticosterone, the influence of a daily 15-min early handling procedure on dams spontaneous behavior, retrieval, nursing (arched-back, blanket, passive), pup care (licking, anogenital licking, digging), dam self-care (self-grooming, eating, drinking), and other behaviors (rearing, moving, resting, standing out of nest) were scored. Results from a two-way ANOVA performed on MB-I as dependent variable and prenatal corticosterone and early handling as independent variables, showed that: the factor prenatal corticosterone (*F*_(1,76)_ = 19.77; *p* < 0.0001) early handling (*F*_(1,76)_ = 64.58; *p* < 0.0001) and their interaction (*F*_(1,76)_ = 8.646; *p* = 0.0043) were significant. In detail, *post hoc* analysis conducted by Tukey’s multiple comparison post-test highlighted a significant lower maternal behavior in Ct treated dams (*q* = 7.386, *p* < 0.0001) with respect to Vh group. Moreover, early handling was able to increase dams maternal behavior in both Vh-H (*q* = 5.096, *p* < 0.0031) and Ct-H (*q* = 10.98, *p* < 0.0001) groups, when compared to respective control groups (Figure 2).

### Open Field Test

**Figure 3:**
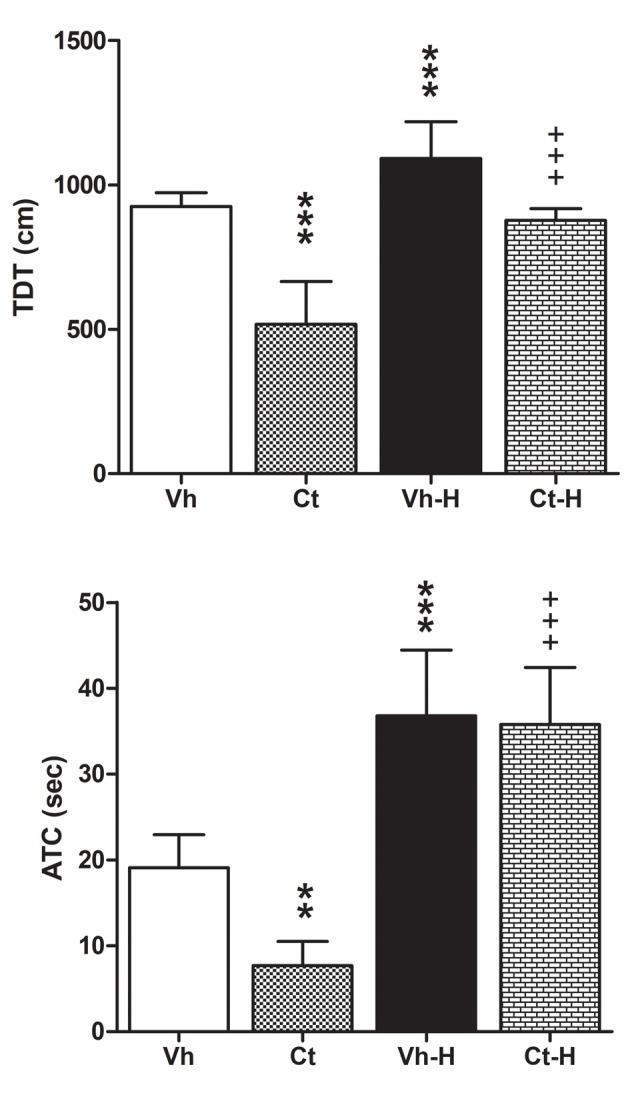
Open field test (OFT). Effects of prenatal corticosterone exposure and early handling procedure on total distance traveled (TDT), and amount of time spent (ATC) in the central area of the arena. Each value represents the mean ± SD of 10 rats. ***p < 0.001, **p < 0.01, vs. Vh, ^+++^*p* < 0.001 vs. Ct.

Rats were tested in the OFT in order to assess the influence of prenatal corticosterone exposure and early handling on behavioral reactivity. Results obtained by a two-way ANOVA performed on total distance travelled, number of transition from peripheral to central squares of the arena, and amount of time spent on the central areas as dependent variables, and prenatal corticosterone and early handling as independent variables, showed that prenatal corticosterone, early handling and their interaction were significant for TDT (*F*_(1,36)_ = 91.79, *p* < 0.0001; *F*_(1,36)_ = 66.16, *p* < 0.0001; *F*_(1,36)_ = 8.866, *p* = 0.0052), and ATC (*F*_(1,36)_ = 6.388; *p* = 0.0160; *F*_(1,36)_ = 87.15; *p* < 0.0001; *F*_(1,36)_ = 4.494; *p* = 0.0410). *Post hoc* analysis conducted by Tukey’s multiple comparison post-test showed that prenatal Ct induced a decrease in both TDT and in ATC (*q* = 12.56; *p* < 0.001; *q* = 4.647; *p* < 0.0116) when compared to Vh groups. On the contrary, the early handling procedure induced an increase in TDT and in ATC in both Vh (*q* = 5.156, *p* < 0.0044; *q* = 7.216, *p* < 0.001) and Ct (*q* = 17.71, *p* < 0.001; = 11.46, *p* < 0.001) treated rats when compared to respective controls (Figure 3). No statistical difference was observed when Ct-H was compared to Vh-H group. No statistical differences were found on a number of transitions from peripheral to central squares of the arena.

### Acoustic Startle Reflex Test

**Figure 4:**
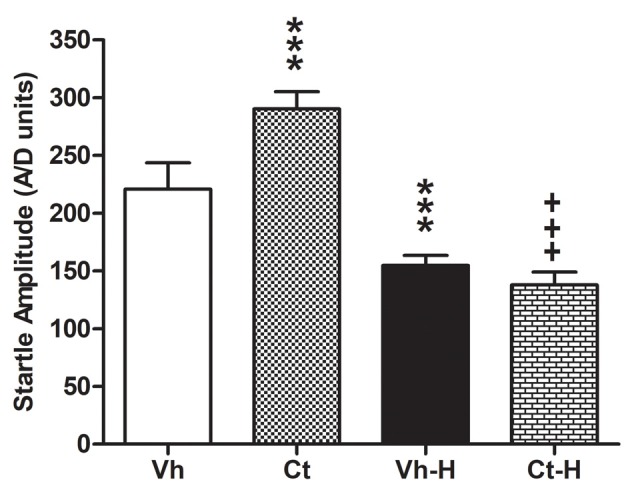
Effects of prenatal corticosterone exposure and early handling procedure on the peak amplitude in acoustic startle reflex (ASR). Each value represents the mean ± SD of 10 rats. ***p < 0.001 vs. Vh, ^+++^*p* < 0.001 vs. Ct.

The effects of prenatal corticosterone exposure and the influence of early handling procedure on the response to an anxiety-inducing intense stimulus, were evaluated measuring startle amplitude in the ASR test. The results of a two-way ANOVA performed on the peak amplitude as dependent variable, and prenatal corticosterone and early handling as independent variables, indicated that: the factor prenatal corticosterone (*F*_(1,36)_ = 29.48; *p* < 0.0001) early handling (*F*_(1,36)_ = 503.4; *p* < 0.0001) and their interaction (*F*_(1,36)_ = 78.57; *p* < 0.0001) were significant. In detail, Tukey’s multiple comparison post-test analysis showed that the prenatal treatment with Ct induced an increase in startle amplitude (*q* = 14.29; *p* < 0.001) when compared to Vh. Interestingly, early handling was able to reduce startle amplitude in both Vh (*q* = 13.57; *p* < 0.001) and in Ct (*q* = 31.30; *p* < 0.001) treated rats, when compared to respective controls. No statistical difference was observed when Ct-H was compared to Vh-H group (*q* = 16.70; *p* > 0.05; Figure 4).

### Forced Swim Test

**Figure 5:**
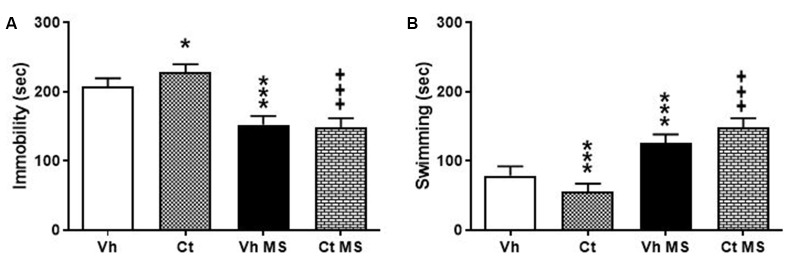
Effects of prenatal corticosterone exposure and early handling procedure on immobility **(A)** and swimming **(B)** in the Forced swim test (FST). Each value represents the mean ± SD of 10 rats. ***p < 0.001, *p < 0.05 vs. Vh, ^+++^*p* < 0.001 vs. Ct.

Rats were tested in the Porsolt test in order to evaluate the effects of prenatal exposure to corticosterone and the influence of early handling procedure on depressive-like behavior. Rats were first exposed to the pre-stage and, 24 h after, underwent the 5-min stage test, when immobility-, swimming-time were recorded. A two-way ANOVA performed on time spent in immobility, swimming, as a dependent variable, and prenatal corticosterone and early handling as independent variables. The results indicate that prenatal corticosterone, early handling and their interaction were significant for both immobility (*F*_(1,36)_ = 5.327; *p* = 0.0269); (*F*_(1,36)_ = 299.9; *p* < 0.0001); (*F*_(1,36)_ = 10.81; *p* = 0.0023) and swimming (*F*_(1,36)_ = 7.119; *p* = 0.0114); (*F*_(1,36)_ = 242.9; *p* < 0.0001); (*F*_(1,36)_ = 11.32; *p* = 0.0018). Tukey’s multiple comparison post-test analysis showed that the prenatal treatment with corticosterone induced an increase in immobility time in Ct (*q* = 5.596; *p* < 0.0019) with respect to Vh, and a significant decrease on immobility time in both Vh-H (*q* = 14.03; *p* < 0.001) and in Ct-H (*q* = 20.6; *p* < 0.001) when compared respectively with Vh and Ct groups (Figure 5A). In agreement with these results, *post hoc* analysis showed a significant decrease in Ct (*q* = 6.033; *p* = 0.0008) compared to Vh-rats and an increase in swimming time in both Vh-H (*q* = 12.22; *p* < 0.001) and in Ct-H (*q* = 18.95; *p* < 0.001) with respect to their non-handled controls, and (Figure 5B).

### Corticosterone Plasma Levels

**Figure 6:**
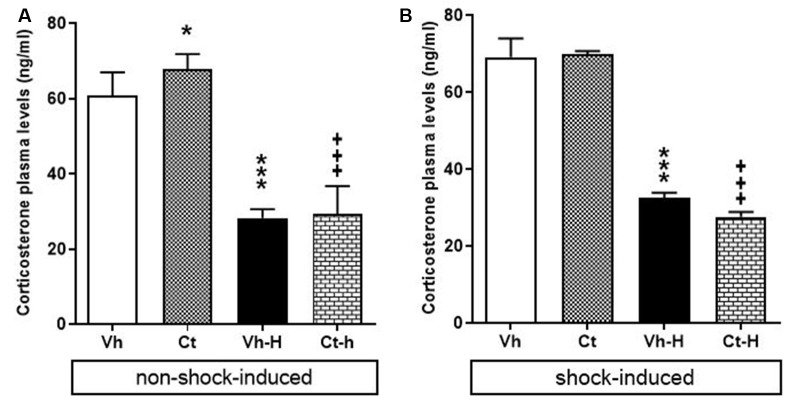
Effects of prenatal corticosterone exposure and early handling procedure on: non-shock- **(A)** and shock-induced **(B)** corticosterone plasma levels. Each value represents the mean ± SD of five rats. ***p < 0.001, *p < 0.05 vs. Vh, ^+++^*p* < 0.001 vs. Ct.

The effects of prenatal exposure to corticosterone, early handling and their mutual influence on corticosterone plasma levels in rats under non-shock- or shock-induced stress conditions were also investigated. A two-way ANOVA performed on the levels of corticosterone under non-shock-induced conditions as dependent variables, and prenatal corticosterone treatment, early handling as independent variables indicate that: prenatal corticosterone (*F*_(1,36)_ = 5.311; *p* = 0.0271) early handling (*F*_(1,36)_ = 515.9; *p* < 0.0001) and their interaction (*F*_(1,36)_ = 4.502; *p* = 0.004), were significant under non-shock-induced conditions. The results of Tukey’s multiple comparisons test showed that non-shock-induced corticosterone plasma levels increased in Ct-exposed offspring compared to vehicle group (*q* = 4.426; *p* < 0.0174) and that early handling reduced corticosterone plasma levels in both Vh-H (*q* = 20.59, *p* < 0.0001; *q* = 24.84, *p* < 0.0001) and in Ct-H (*q* = 26.23, *p* < 0.001; *q* = 28.67, *p* < 0.001) when compared with respective control groups (Figure 6A).

When rats were exposed to shock-induced stress conditions in order to evaluate the corticosterone plasma levels under stressful conditions, the results of a two-way ANOVA performed respectively on the levels of corticosterone as dependent variables, and prenatal corticosterone treatment, early handling as independent variables showed a significant effect for early handling (*F*_(1,36)_ = 913.3; *p* < 0.0001) and interaction between corticosterone treatment and early handling (*F*_(1,36)_ = 4.325; *p* = 0.0447), but no for prenatal corticosterone treatment (*F*_(1,36)_ = 2.584; *p* = 0.1167). In detail, Tukey’s multiple comparison post-test analysis showed that; shock exposure did not modify corticosterone levels in Ct-exposed offspring (*q* = 0.472, *p* = 0.9870), and that early handling reduced corticosterone plasma levels in both Vh-H (*q* = 28.14, *p* < 0.0001) and in Ct-H (*q* = 32.30, *p* < 0.0001) when compared with respective control groups (Figure 6B).

## Discussion

In agreement with previous animal studies, we here show that exposure to corticosterone, during the 3rd week of rat gestation, can affect maternal care and program an abnormal neuroendocrine and behavioral profile of the adolescent offspring that resembles a vulnerable phenotype for affective disorders (French et al., 1999; Shoener et al., 2006). Notably, early handling as a brief maternal separation during the early stages of postnatal life, promoted an increase in maternal care and counterbalanced the detrimental effects induced by the prenatal glucocorticoid manipulation in all the investigated parameters.

### Effects of Prenatal Exposure to Corticosterone

The first evidence following the manipulation of the intrauterine environment by corticosterone injection from GD 14 to 16 was a reduction in weight during the pre-weaning time. Our data are in accordance with studies showing that glucocorticoid treatment during pregnancy reduces offspring birth weight and body weight throughout adolescence (Smith and Waddell, 2000; Manojlović-Stojanoski et al., 2012) as well as the reduction on birth weight appears more evident when glucocorticoids are administered during the 3rd week of gestation and not earlier, indicating a late gestational window of sensitivity to glucocorticoids (Nyirenda et al., 1998; Seckl, 2004). Although the reduced body weight of the offspring as a consequence of gestational corticosterone exposure is still not fully clear, Iwasa et al. (2014) suggested a possible alteration of serum leptin and hypothalamic neuropeptide Y (NPY) mRNA levels, two peptides playing pivotal roles in the regulation of appetite and calories intake, as well as in the modulation of emotionality (Velísek, 2006; Iwasa et al., 2014; Plescia et al., 2014a).

A critical outcome of glucocorticoid exposure in early life is the programming of emotional and affective homeostasis. In the rat, *in utero* glucocorticoids, either from an exogenous source or *via* maternal extra-release, induce a decrease in behavioral reactivity in the open field and an increase in anxiety-like behavior in the elevated plus-maze in the offspring (Harris and Seckl, 2011). These alterations may be associated with an impairment in offspring’s capacity to cope under a stressful situation in adolescence (Vallée et al., 1997; Dickerson et al., 2005; Harris and Seckl, 2011), enhancing the risk of emerging psychological disorders (Casey et al., 2010). Accordingly, our data demonstrate that the prenatal Ct-treatment during a sensitive time window, was able to induce an overall impairment of locomotor activity in the adolescent offspring, as shown by a reduction in TDT and in the exploration of the central areas of the arena. The reduction in behavioral reactivity might reflect an increased emotional response to the novel environment. Consistently, adolescent rats exposed *in utero* to corticosterone exhibited an increase in the peak amplitude of the ASR, as a proof of their negative emotional state (Lang et al., 1990; Lang, 1995; Bradley and Sabatinelli, 2003; McMillan et al., 2012). Indeed, The ASR, a reflexive movement occurring after sudden exposure to loud noise, represents a valid behavioral model to study the emotional response of the animals. An increase in the amplitude of the ASR is ascribed to a rise in emotionality, which mirrors a higher sensitivity of the animals towards an anxiogenic environment (Hijzen et al., 1995; Cannizzaro et al., 2002). These results are in accordance with our data on the FST the most commonly used assay to test the efficacy of chronic antidepressant treatments (Detke et al., 1997). Our findings indicate that prenatal Ct treatment was able to increase immobility time and decrease swimming in the adolescent offspring, promoting the occurrence of a depressive-like phenotype (Yankelevitch-Yahav et al., 2015).

Indeed, over-exposure to glucocorticoids and impaired GR signaling can result in degeneration and functional impairment of brain regions critically involved in mood processing and contribute to the induction of depressive symptoms later in life (Anacker et al., 2011; Brancato et al., 2017; Di Liberto et al., 2017; Shishkina and Dygalo, 2017).

During development, there is a relatively high expression of GR from midgestation onwards (Diaz et al., 1998), which are essential for normal brain development and offspring survival (Kapoor et al., 2008). In the rat, antenatal stress or maternal administration of glucocorticoids during this time window results in offspring with decreased expression of GR mRNA in specific brain areas involved in glucocorticoid feedback such as the hippocampus, hypothalamus, and pituitary (Levitt et al., 1996; Liu et al., 2001). This reduction could promote pups grow up with altered negative feedback response, manifested as a chronic elevation of corticosterone (Maccari and Morley-Fletcher, 2007). Indeed, the behavioral outcomes here observed are supported by the results from plasma corticosterone level assessment in non-shock-induced conditions. Specifically, prenatally exposed adolescent offspring showed an increase in non-shock-induced plasma corticosterone levels, in line with findings in rodents and non-human primates (Welberg et al., 2001; de Vries et al., 2007; Rakers et al., 2017). It has been shown previously that differences in HPA axis activity are associated with differences in locomotor activity in response to novelty (Gancarz et al., 2012). Prenatal stress induces a prolonged corticosterone secretion, which is negatively correlated with lower levels of explorative behavior in the open field (Rosecrans, 1970; Iuvone and Van Hartesveldt, 1976; Vallée et al., 1997). Moreover, a significant correlation between plasma corticosterone levels and the behavioral scores in the FST was observed (Morley-Fletcher et al., 2003).

Plasma corticosterone levels in the non-shock-induced group do not differ from levels in the shock-induced group. This may be for that non-shocked group plasma corticosterone levels do not reflect baseline activity of the HPA axis, but rather HPA axis reactivity in response to novelty of the electrified grid floor cage (Friedman et al., 1967; Bassett et al., 1973). Furthermore, differently from plasma corticosterone levels in shock-induced condition, we found that corticosterone release after shock administration did not differ between prenatally exposed adolescent offspring and Vh group. This can be due to an altered drive of the HPA axis programming that may result from the combination of *in utero* Ct-treatment and stress exposure in adolescence. Indeed, we may speculate that prenatal corticosterone treatment was able to reduce the density of corticosteroid receptors that, through the attenuation of HPA axis feedback sensitivity, set the release of corticosterone to a ceiling set point already at basal conditions (Pornsawad, 2013). This might prevent the physiological rise in stress-related glucocorticoid release as we have observed in this study and might represent a vulnerable factor for the development of emotional and affective disorders (Harris and Seckl, 2011; Constantinof et al., 2016).

### Effects of Early Brief Maternal Separation

In most mammalian species, the maternal environment represents the developmental context within which mothers shape socio-emotional maturation of the progeny, serving as essential external regulators of infant physiology, neurodevelopment, and behavioral responses. Thus, manipulating quality and consistency of maternal care during the early stages of life can influence and, also revert developmental processes that set emotional and physiological responses in adulthood (Drury et al., 2016).

Numerous studies have shown that at least some of the long-term effects of early-life exposure to an adverse environment are mediated by low levels of parent-child linking and decreased parental investment during early childhood. For instance, poor parental ties are usually associated with increased risk for several psychological vulnerabilities, whereas an increase in parental care improved behavioral outcomes, cognitive performance and also boost resiliency to stress (Canetti et al., 1997; Meaney, 2001; Kaffman and Meaney, 2007). Accordingly, early handling procedure, consisting in a short maternal separation of the mother from the pups, represents a particular event for the dam that is able to produce higher level of interest by the mother in the offspring and, in turn, elicits more maternal care upon reunion (Rees and Fleming, 2001; Kosten and Kehoe, 2010; Zimmerberg and Sageser, 2011; Own and Patel, 2013; Orso et al., 2018). These observations are consistent with those obtained in the present research where the effects on the maternal-infant dyad were investigated. Indeed, our results show that early handling procedure produced an increase in maternal care, as shown by a higher MB-I, that in turn, improved the response to stressful situations and reduced emotionality in the offspring. Specifically, when compared to non-handled counterparts, briefly maternal separated adolescent rats showed increased locomotor activity, reduced avoidance of the center of the arena in the open field, and decreased peak amplitude in ASR. At the same time, early-handled offspring displayed a reduction in immobility time and an increase in swimming time in the FST, together with a reduction in corticosterone plasma levels, under non-shock- and shock-induced conditions.

The mitigated emotional profile observed in early handled rats in this study may be dependent upon modifications of the developing HPA axis (Kaffman and Meaney, 2007). In particular, the effect of early handling on behavioral reactivity and emotionality may be due to a dampening of HPA axis response in the progeny that better cope with the task administered (Cannizzaro et al., 2006b). Indeed, maternal behaviors, such as licking, grooming and arched-back, lead to increased GR mRNA expression in the brain, glucocorticoid negative feedback sensitivity, and decreased hypothalamic corticotropin-releasing factor mRNA levels (Meaney, 2001; Edelmann et al., 2016). Taken together these data suggest that postnatal maternal care is able to affect the magnitude of the HPA axis response to stress, “hardening” the pups which display a blunting in corticosterone release and in emotional profile (Meaney et al., 1985, 1988; Liu et al., 1997). On the other hand, the variations in the early postnatal environment can interact with the effects of prenatal exposure to stressors in a complex, mutually interacting process (Cannizzaro et al., 2006b). Indeed, whether early exposure to corticosterone is associated with elevation of non-shock-induced conditions corticosterone release and with a vulnerable phenotype for emotional and affective disturbances, early handling procedure induces opposite modifications in the stress-behavioral responses and corticosterone release that are associated to the occurrence of a “rescued” profile. Although we believe that the rodent model used in this study will be helpful to identify physiological mechanisms underlying the neuroendocrine functional response to stress induced by early handling in prenatal corticosterone condition, this issue deserves further insight in future researches on many distinct players which may take part to the interplay between maternal care and the regulation of the HPA axis, such as oxytocin (Cannizzaro et al., 2006a; Kojima et al., 2012; Cox et al., 2015; Zinni et al., 2018). However, it is evident that increasing the intensity of maternal care, could serve as a source for the enhancement of neuronal plasticity able to promote adaptive behavioral responses.

### Conclusion

These findings highlight a brief prenatal exposure to glucocorticoids during the 3rd week of gestation as a signal able to produce behavioral and neuroendocrine abnormalities later in life, contributing to the programming of a vulnerable phenotype to emotional- and affective-like disorders. This issue is particularly relevant due to the common practice of multiple administrations of glucocorticoids to pregnant women during late gestation to ensure the survival of the preterm newborns. Even though synthetic glucocorticoids, such as dexamethasone (DEX) or betamethasone, have been extensively used rather than cortisol or hydrocortisone (Jobe, 2003; Oliveira et al., 2006; Singh et al., 2012), the natural glucocorticoid is increasingly considered as an alternative therapy during pregnancy (Crowther et al., 2019). Therefore, a long-term follow-up in children who were treated *in utero* with glucocorticoids is strongly recommended. As expected, we here show that enhanced maternal care plays a primary role in setting pro-adaptive behavioral and neuroendocrine responses and may re-route aberrant trajectories during neurodevelopment, emphasizing the role of an optimal mother-infant dyad as a protective factor for healthy development of the offspring.

## Data Availability Statement

The datasets generated for this study are available on request to the corresponding author.

## Ethics Statement

The animal study was reviewed and approved by the Committee for the Protection and Use of Animals of the University of Palermo.

## Author Contributions

FP has formulated evolution of overarching research goals and aims and has coordinated the research activity planning and execution. He has provided statistical analyses, has written the article and has acquired the financial support for the project leading to this publication. VC has carried out research and investigation activity, performed the experiments, and has collaborated on the writing of the manuscript. GL and AB has carried out research and investigation activity, performed the experiments.

## Conflict of Interest

The authors declare that the research was conducted in the absence of any commercial or financial relationships that could be construed as a potential conflict of interest.

The reviewer MK declared a shared affiliation, with no collaboration, with one of the authors, GL, to the handling editor at the time of review.
